# Host SAMHD1 protein restricts endogenous reverse transcription of HIV-1 in nondividing macrophages

**DOI:** 10.1186/s12977-018-0452-z

**Published:** 2018-10-13

**Authors:** Bijan Mahboubi, Christina Gavegnano, Dong-Hyun Kim, Raymond F. Schinazi, Baek Kim

**Affiliations:** 1 Center for Drug Discovery, Department of Pediatrics, School of Medicine, Emory University, Atlanta, GA 30322 USA; 2 School of Pharmacy, Kyung-Hee University, Seoul, South Korea; 3 Children’s Healthcare of Atlanta, Atlanta, GA USA

**Keywords:** HIV-1, HIV-2, Reverse transcription, Endogenous reverse transcription, SAMHD1, Vpx, dNTPs, Macrophages

## Abstract

**Background:**

SAM domain and HD domain containing protein 1 (SAMHD1) is a host anti-HIV-1 restriction factor known to suppress viral reverse transcription in nondividing myeloid cells by its dNTP triphosphorylase activity that depletes cellular dNTPs. However, HIV-2 and some SIV strains rapidly replicate in macrophages due to their accessory protein, viral protein X (Vpx), which proteosomally degrades SAMHD1 and elevates dNTP levels. Endogenous reverse transcription (ERT) of retroviruses is the extra-cellular reverse transcription step that partially synthesizes proviral DNAs within cell-free viral particles before the viruses infect new cells. ERT activity utilizes dNTPs co-packaged during budding from the virus-producing cells, and high ERT activity is known to enhance HIV-1 infectivity in nondividing cells. Here, since Vpx elevates cellular dNTP levels in macrophages, we hypothesize that HIV-2 should contain higher ERT activity than HIV-1 in macrophages, and that the Vpx-mediated dNTP elevation should enhance both ERT activity and infectivity of HIV-1 particles produced in macrophages.

**Results:**

Here, we demonstrate that HIV-2 produced from human primary monocyte derived macrophages displays higher ERT activity than HIV-1 produced from macrophages. Also, HIV-1 particles produced from macrophages treated with virus like particles (VLPs) containing Vpx, Vpx (+), displayed large increases of ERT activity with the enhanced copy numbers of early, middle and late reverse transcription products within the viral particles, compared to the viruses produced from macrophages treated with Vpx (−) VLPs. Furthermore, upon the infection with an equal p24 amount to fresh macrophages, the viruses produced from the Vpx (+) VLP treated macrophages demonstrated higher infectivity than the viruses from the Vpx (−) VLP treated macrophages.

**Conclusions:**

This finding identifies the viral ERT step as an additional step of HIV-1 replication cycle that SAMHD1 restricts in nondividing myeloid target cells.

**Electronic supplementary material:**

The online version of this article (10.1186/s12977-018-0452-z) contains supplementary material, which is available to authorized users.

## Background

All lentiviruses including type 1 and type 2 human immunodeficiency virus (HIV-1 and HIV-2) and simian immunodeficiency virus (SIV) infect both activated/dividing CD4^+^ T cells and terminally differentiated/nondividing myeloid cells such as macrophages and microglia during the course of their pathogenesis [1, 2]. However, while HIV-1 rapidly replicates in activated CD4^+^ T cells, HIV-1 replication in nondividing myeloid cells is kinetically suppressed [3–5]. We previously demonstrated that this restricted replication kinetics of HIV-1 in myeloid cells is due to the extremely low dNTP concentrations found in this nondividing cells type, which blocks the viral reverse transcription step that consumes cellular dNTPs during proviral DNA synthesis [6]. A series of recent studies reported that a host dNTPase, sterile alpha motif (SAM) domain and histidine-aspartate (HD) domain containing protein 1 (SAMHD1), which hydrolyzes dNTPs to dNs and triphosphates, is responsible for the poor dNTP availability in macrophages, suggesting that SAMHD1 is a myeloid cell specific host restriction factor against HIV-1 [7, 8].

Unlike HIV-1, HIV-2 and some SIV strains replicate rapidly even in macrophages due to their unique accessary protein, viral protein X (Vpx) [9], which counteracts the anti-viral activity of SAMHD1 [7, 10]. Vpx recruits SAMHD1 to the CRL4 (DCAF1) E3-ligase complex for the proteosomal degradation of SAMHD1, leading to the elevation of cellular dNTP levels and rapid reverse transcription kinetics of these Vpx encoding lentiviruses in macrophages [11, 12]. However, the replication of HIV-1 in activated CD4^+^ T cells is not significantly affected by the dNTPase activity of SAMHD1 because SAMHD1 in dividing cells is phosphorylated at its C-terminal site (Threonine at the residue 592) [13], which blocks the formation of the enzymatically active tetramer form of SAMHD1 [14, 15]. Furthermore, the dNTP biosynthesis, which is closely tied to cell cycle, is active in dividing cells, and cellular dNTPs in activated/dividing CD4^+^ T cells are highly abundant (1–5 μM), compared to nondividing macrophages (20–40 nM) [6], which can support the robust replication kinetics of HIV-1 in this dividing cell type.

In addition to the reverse transcription step of HIV-1, the DNA gap filling step of the HIV-1 integration requires cellular dNTPs. The integration step of HIV-1, which is mediated by virally encoded integrase, leaves 2–3 nucleotide single stranded (ss) DNA gaps at both 5′ sides of the partially integrated double stranded proviral HIV-1 DNAs [16]. These ss DNA gaps can be repaired by host DNA polymerases, which also consume cellular dNTPs. Indeed, recent biochemical studies suggested that the HIV-1 DNA gap repair is dependent on the cellular dNTP availability and SAMHD1-mediated dNTP depletion can kinetically delay the gap filling reaction during viral integration process in nondividing cells [17, 18].

Mature HIV-1 particles can initiate reverse transcription even before infection if dNTP substrates are available within viral particles. Indeed, it was previously reported that cell free mature HIV-1 particles contain partially synthesized proviral DNAs even before they infect cells, suggesting that the viral cores contain dNTPs and these dNTP are likely co-packaged during virus budding. This cell-free reverse transcription of HIV-1 is called endogenous reverse transcription (ERT) [19]. With this ERT activity, HIV-1 can proceed proviral DNA synthesis through not only the early 1st strong-stop DNA product but also intermediate/late (−) strand DNA products before infection. Furthermore, HIV-1 particles with high ERT activity, which already initiated the proviral DNA synthesis, display higher viral infectivity particularly in macrophages [20] likely because these viruses already completed some of the reverse transcription steps that is rate limiting in this nondividing cell type due to the SAMHD1-mediated dNTP depletion.

In this study, since the Vpx-induced dNTP elevation in macrophages can allow the budding viral particles to co-package more dNTPs, we hypothesize that HIV-2 produced from macrophages should have higher ERT activity than HIV-1 produced from macrophages. We further hypothesize that Vpx can enhance the ERT activity of HIV-1 in macrophages and the infectivity of the produced viruses to new macrophages. Indeed, this study demonstrates that HIV-2 harbors higher ERT activity than HIV-1 in macrophages, and that Vpx was able to increase both ERT activity and infectivity of HIV-1 in macrophages. These findings support that the higher ERT activity of HIV-2 may contribute to its efficient replication kinetics in macrophages, and that the ERT step is an additional step of HIV-1 replication cycle that is restricted by host SAMHD1 protein in nondividing myeloid cells.

## Results

### ERT activity comparison between HIV-1 and HIV-2 produced from human primary monocyte derived macrophages

**Fig. 1:**
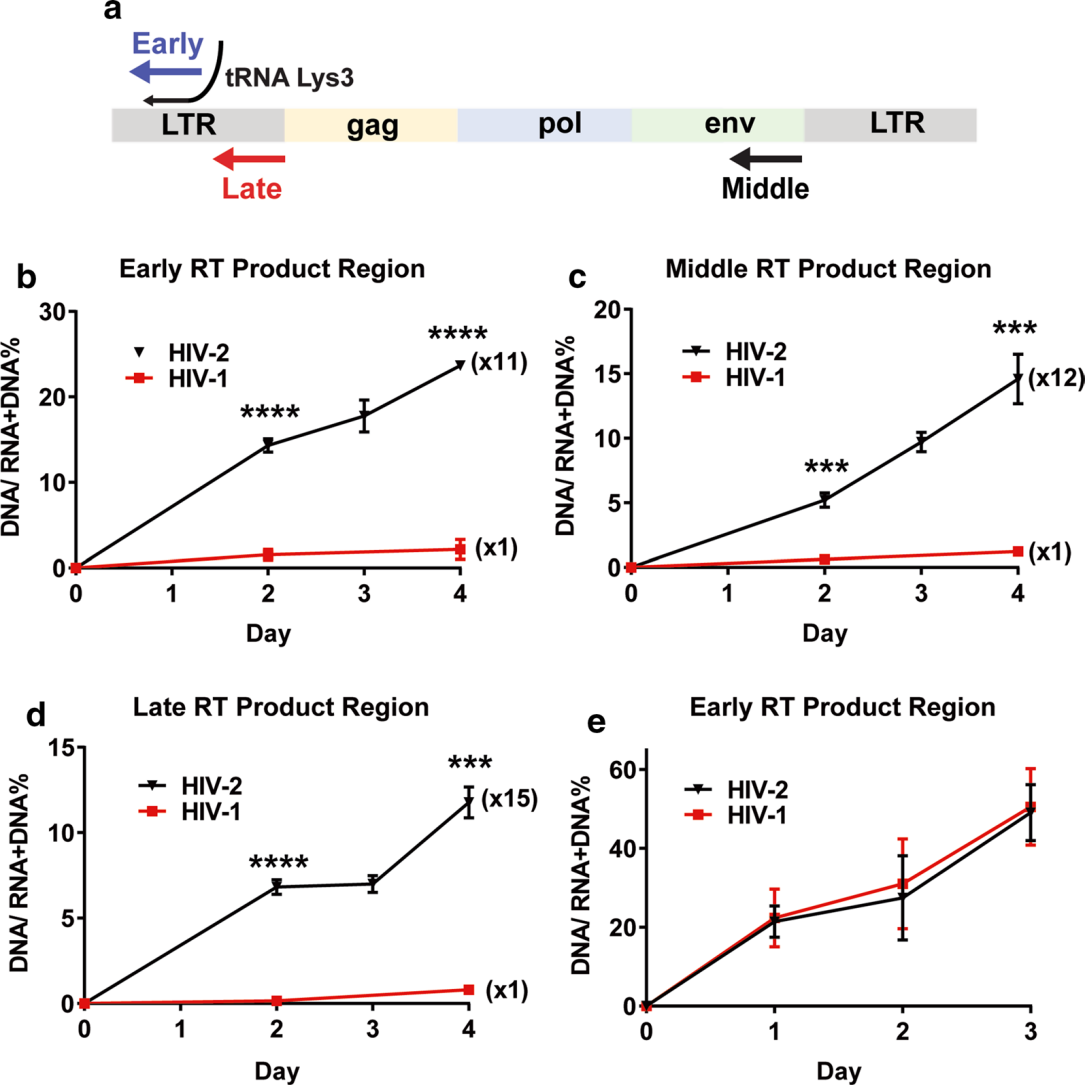
ERT activity comparison between HIV-1 and HIV-2 produced from human primary monocyte-derived macrophages and activated CD4 + T cells. **a** Three RT product regions (early, middle and late) of HIV-1 and HIV-2 genomes used for measuring viral endogenous reverse transcription (ERT) activity. **b**–**d** ERT activities of HIV-1 89.6 and HIV-2 Rod10 produced from macrophages. Human primary monocyte derived macrophages from 5 healthy donors were infected with HIV-1 89.6 (red line) and HIV-2 Rod10 (black line) in triplicates, and the remaining uninfected viruses were extensively washed at 9 h post infection. The media containing the produced viruses were collected at days 2, 3 and 4 post infection, and the total viral nucleic acids of the produced viral particles were extracted for the Q-RT PCR (for RNA + DNA) and Q-PCR (for DNA only) for the early (**b**), middle (**c**), and late regions (**d**) of the viral genomes. ERT activity assay was determined in triplicates by the ratio between DNA copy number and (RNA + DNA) copy number in the produced viral particles. Fold differences between ERT activities of HIV-1 89.6 (1 ×) and HIV-2 Rod10 were calculated. **e** ERT activity of HIV-1 89.6 and HIV-2 Rod10 produced from activated CD4 + T cells isolated from the same donors. ERT assay was conducted for the early region with the viruses produced up to 3 days. The data are the mean of three independent experiments with qPCR performed in duplicate, and error bars represent the standard error of the mean. *P < 0.05; **P < 0.01; ***P < 0.001; ****P < 0.0001

Retroviruses co-package cellular dNTPs from virus producing cells during viral assembly and these co-packaged dNTPs are used by reverse transcriptase (RT) even before the viruses infect new cells. This cell-free reverse transcription process is called endogenous RT (ERT) activity, which enhances viral infected particularly to the non-dividing cells that restricts reverse transcription kinetics due to limited cellular dNTPs [20]. HIV-2 Vpx elevates cellular dNTP pools in the infected macrophages, and therefore, it is likely that Vpx-induced dNTP elevation in macrophages can allow the budding viral particles to co-package more dNTPs, compared to HIV-1. Here, we hypothesize that HIV-2 produced from macrophages should harbor higher ERT activity than HIV-1 produced from macrophages. To test this hypothesis, we infected human primary monocyte-derived macrophages (from 5 healthy donors) with HIV-1 89.6 and HIV-2 Rod10, and measured the ERT activity of the produced viruses. The ERT activity is determined by the ratio between copy number of viral DNAs and copy number of (RNA + DNA) contained in the cell free viral particles produced from the infected cells [20]. The higher DNA versus (RNA + DNA) copy ratio will indicate the higher ERT activity. For the ERT assay, Q-RT-PCR for measuring copy numbers of both viral RNAs and viral DNAs and Q-PCR for determining only the copy numbers of viral DNAs were conducted with the total viral nucleic acids isolated from the produced viral particles. As shown in Fig. 1a, three regions of viral genome, (1) early region encoding the 1st strong-stop DNA, (2) middle region encoding near the end of the env gene, and (3) late region encoding the sequences at the downstream of primer binding site (PBS), were analyzed for the ERT activity of the produced viral particles.

Primary monocyte derived macrophages infected with HIV-1 89.6 or HIV-2 Rod10 were washed to remove the remaining uninfected viruses, and then the culture media were collected at days 2, 3, and 4 post infection for the isolation of the total viral nucleic acids. The ERT activity of HIV-1 89.6 and HIV-2 Rod10 for the three different regions of the viral gene were determined. Indeed, as shown in Fig. 1b–d, HIV-2 Rod10 displayed 11-15 times higher ERT activity in all three regions than HIV-1 89.6 produced from macrophages. We also infected activated CD4^+^ T cells isolated from the same donors with the same amount of HIV-1 89.6 and HIV-2 Rod10 as used for the macrophage infection. For activated CD4^+^ T cells, the infected cells were cultured for 3 days and the produced viral particles were collected at days 1, 2 and 3 post infection for the ERT activity assay. As shown in Fig. 1e, unlike the viruses produced from macrophages, both HIV-1 89.6 and HIV-2 Rod10 showed very similar ERT activity in activated CD4 + T cells that harbor abundant cellular dNTPs. The data presented in Fig. 1 demonstrates that HIV-2 has higher ERT activity than HIV-1 in nondividing macrophages, which is likely due to Vpx that elevates cellular dNTPs and then enhance the co-packaging of cellular dNTPs into the produced HIV-2 viral particles.

### ERT activity of HIV-1 89.6 in activated CD4^+^ T cells and macrophages with and without Vpx treatment

**Fig. 2:**
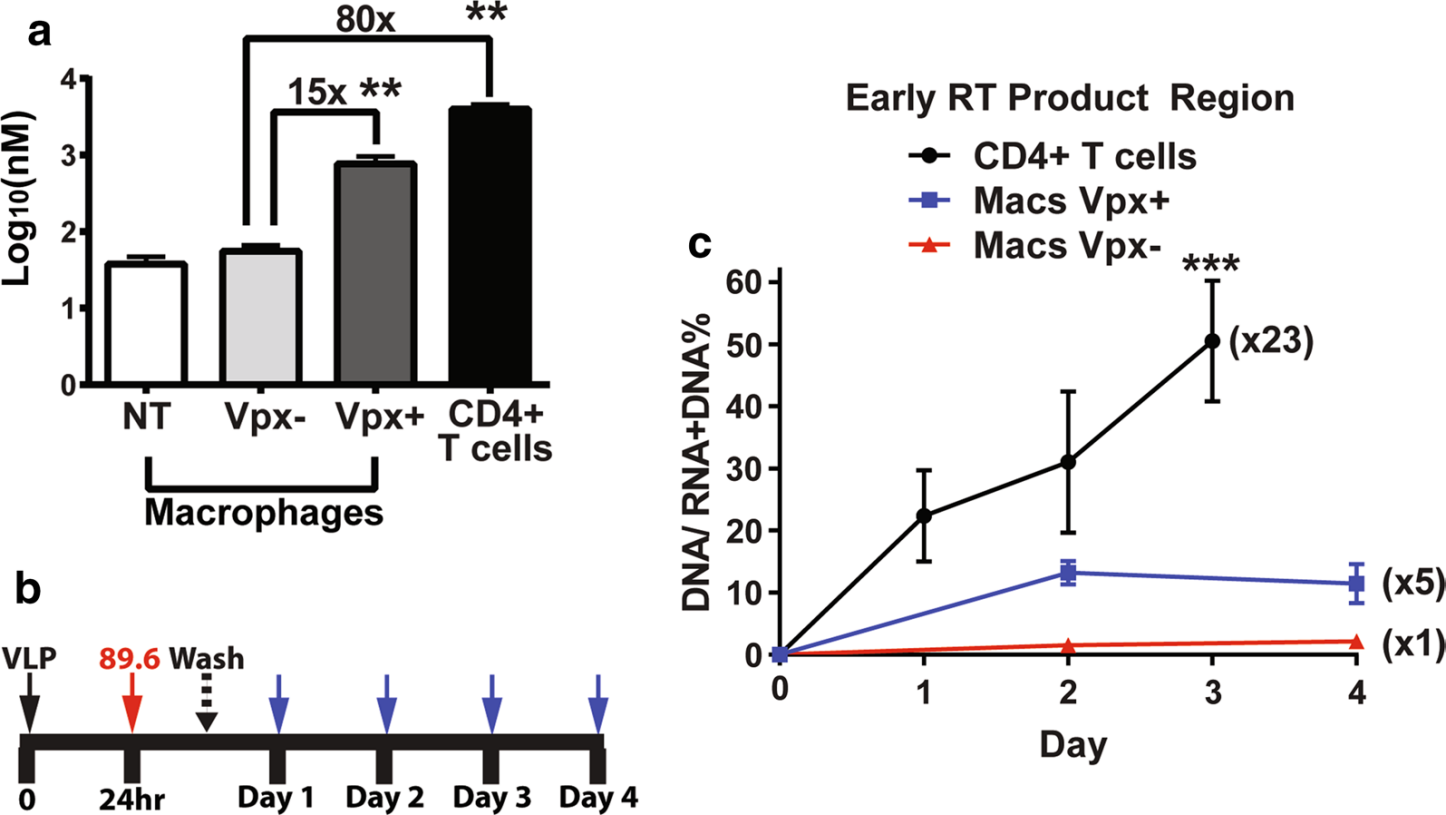
ERT activity comparison of HIV-1 89.6 produced from macrophages treated with Vpx (−) and Vpx (+) virus like particles. **a** Comparison of cellular dATP concentrations among Vpx (−) virus like particles (VLPs) treated macrophages, Vpx (+) VLP treated macrophages, and activated CD4 + T cells. Human primary monocyte derived macrophages from 5 healthy donors were treated with Vpx (−) and Vpx (+) VLPs as well as without VLP treatment (NT) for 24 h, and cellular dNTPs were extracted from the cells for the RT-based dNTP assay. The dNTP concentrations were calculated based on their cell volumes. The dNTP concentration from activated CD4 + T cells were also determined, and the fold differences of the dNTP concentrations were calculated. Other three dNTP concentrations are shown in Additional file 1: Figure S1A. The VLP treatment and dNTP assay were conducted in triplicates. **b** Protocol for virus collection produced from macrophages for ERT assay. Human primary monocyte-derived macrophages were pre-treated with Vpx (−) and Vpx (+) VLPs for 24 h, and then infected with HIV-1 89.6 in triplicates. The remaining uninfected viruses were washed at 9 h post infection, and the viruses produced from these cells were collected for the ERT activity measurement at every 24 h for 4 days. The SAMHD1 degradation in these VLP treated macrophages were confirmed by western blots (Additional file 1: Figure S1B). **c** ERT activity of HIV-1 89.6 produced from macrophages treated with Vpx (−) (red line) and Vpx (+) (blue line) VLPs. ERT activity of the produced viruses were determined as described in Fig. 1. The ERT activity of HIV-1 89.6 from activated CD4 + T cells (black line) was used for the comparison. The data are the mean of three independent experiments with qPCR or dNTP assay performed in duplicate, and error bars represent the standard error of the mean. *P < 0.05; **P < 0.01; ***P < 0.001

Next, we tested the hypothesis that the treatment of macrophages with Vpx can enhance the ERT activity of HIV-1. First, we confirmed the anti-SAMHD1 activity and dNTP elevation activity of Vpx in macrophages isolated from healthy donors. For this, human monocyte derived macrophages were prepared by the differentiation of monocytes isolated and pooled from 5 donors in an equal number. The 7-day differentiated macrophages were treated with virus like particles (VLPs) with (+) or without (−) Vpx for 24 h, and then both SAMHD1 level and dNTP level of the treated cells were determined. As shown in Fig. 1a, the Vpx-treated macrophages (“+”), which displayed significantly reduced level of SAMHD1 protein, compared to the untreated and Vpx (−) VLP treated macrophages (Additional file 1: Figure S1A), showed 10–15 times (15 × for dATP: Fig. 2a) higher dNTP concentrations (200–700 nM) than those of the Vpx (−) VLP treated macrophages and non-treated (“NT”) (20–60 nM). Also, the activated CD4^+^ T cells from the same donors showed 80–100 times higher dNTP concentration (3–5 μM: 80 × for dATP, Fig. 2a) than macrophages treated Vpx (−) VLPs, and 5–8 times higher dNTP concentration than the Vpx (+) VLP treated macrophages. Other three dNTP concentrations (dGTP, dCTP and dTTP) in these tested cells are shown in Additional file 1: Figure S1A.

To test the hypothesis that the treatment of macrophages with Vpx can enhance the ERT activity of HIV-1, as illustrated in Fig. 2b, we pre-treated macrophages from 5 donors with Vpx (−) and Vpx (+) VLPs for 24 h, and then infected cells with HIV-1 89.6. The remaining uninfected viruses in the media were extensively washed at 9 h post infection, and the culture media containing the produced viral particles were collected at every 24 h for 4 days. The total nucleic acids extracted from the collected media were applied for the ERT assay for the early region. As shown in Fig. 2c, the Vpx treatment of macrophages enhanced the ERT activity of HIV-1 89.6 by 5 folds. HIV-1 89.6 harvested from activated CD4^+^ T cells at day 3 showed 23 times higher ERT activity than the viruses harvested at day 4 from macrophages treated with Vpx (−) VLPs (Fig. 2c). The order of ERT activities of the viruses analyzed for the early region is: activated CD4^+^ T cells > Vpx (+) VLP-treated macrophages > Vpx (−) VLP-treated macrophages. Importantly, the order of the ERT activity among the viral particles produced from these three conditions is correlated with the order of the cellular dNTP concentrations found in these three conditions (Fig. 2a): activated CD4^+^ T cells (3–5 µM) > Vpx (+) VLP treated macrophages (200–700 nM) > Vpx (−) VLP treated macrophages (20–60 nM). However, the ERT activity for the middle and late regions were not detected, presumably because the detection of the middle and late products may need longer virus culture.

### Effect of Vpx on the ERT activity during long-term culture of macrophages repeatedly treated with Vpx

**Fig. 3:**
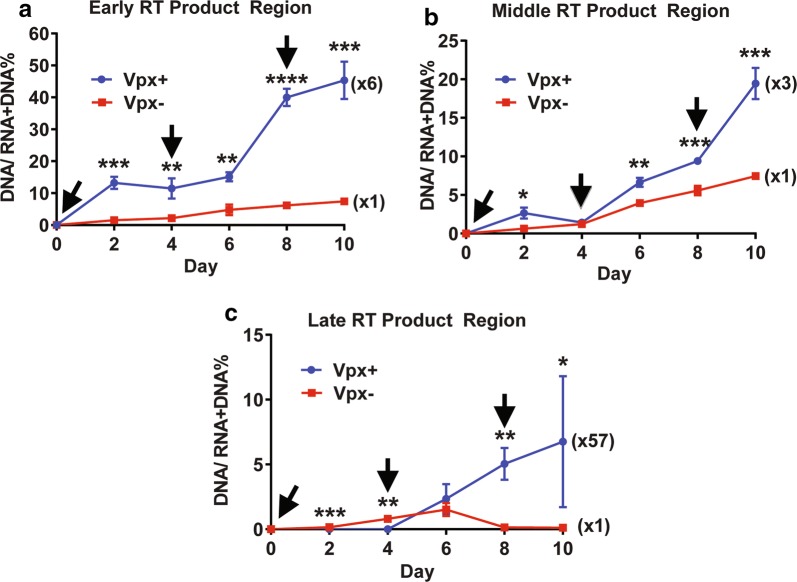
ERT activity comparison between HIV-1 89.6 viruses produced during long-term culture of macrophages with multiple treatments of Vpx (−) and Vpx (+) VLPs. Primary macrophages were pre-treated with Vpx (−) or Vpx (+) VLPs for 24 h, and then infected with an equal p24 level of dual tropic HIV-1 89.6 in triplicates. The remaining uninfected viruses were washed at 9 h post infection. The infected macrophages were cultured for 10 days post infection. During the 10-day culture, the cells were repeatedly treated with Vpx (−) or Vpx (+) VLPs in every 4 days (see black arrows). The produced viruses were harvested at every 2 days, and total viral nucleic acids were extracted and used for measuring the ERT activity of the viral particles produced from Vpx (−) (red line) and Vpx (+) (blue line) VLP treated macrophages for **a** early, **b** middle, and **c** late RT products. The data are the mean of three independent experiments with qPCR performed in duplicate, and error bars represent the standard error of the mean. *P < 0.05; **P < 0.01; ***P < 0.001

In order to compare the ERT activity between the viruses produced from Vpx (−) and Vpx (+) VLP treated macrophages by using the middle and late regions, we analyzed the ERT activity of the viruses harvested during a long-term culture of macrophages up to 10 days. However, we previously reported that the Vpx treatment in macrophages maintains the high dNTP levels only for 2–4 days and then the dNTP level decreases because SAMHD1 starts re-appearing from 5 to 7 days post the Vpx (+) VLP treatment [21]. However, the repeated multiple treatments of macrophages with Vpx (+) VLPs during the long-term culture enabled the cells to maintain the elevated dNTP levels for a longer period of time (> 10 days) [21]. Therefore, we tested whether the viruses produced from macrophages repeatedly treated with Vpx (+) VLPs can maintain high ERT activity during the long-term culture and whether the predicted ERT activity difference can be seen even with the middle and late regions of the viral genome. For this test, we initially pre-treated monocyte-derived macrophages pooled from 5 donors with Vpx (−) and Vpx (+) VLPs and then infected the cells with HIV-1 89.6 at 24 h post the VLP treatment. These cells were re-treated with Vpx (−) and Vpx (+) VLP in every 4 days for 10 days (see black arrows in Fig. 3), and the produced viruses in the media were collected at various time points during this long-term culture. The total nucleic acids in the collected viruses were isolated for the ERT activity for the three regions of HIV-1 genome: (a) early, (b) middle, and (c) late regions. As shown in Fig. 3, the viruses produced from the macrophages repeatedly treated with Vpx (+) VLPs were able to maintain the high ERT activity in the RT products of all three regions, and the fold increase of the ERT activity by the Vpx (+) VLP treatment also remained high throughout this long-term culture. Interestingly, there was a 6-fold increase of early RT product in viral particles collected from the Vpx (+) VLP treated macrophages than viruses produced from the Vpx (−) VLP treated macrophages on day 10. More interestingly, as shown in Fig. 3c, while almost no viruses (~ 0.1%) produced from the Vpx (−) VLP treated macrophages contain the completed late RT product, 2–10% of the viruses (x57) from the Vpx (+) VLP treated macrophages contain the late RT product. Next, we validated the Vpx effect on the HIV-1 ERT activity by employing another HIV-1 strain, BaL, under the same experimental setting used for 89.6. Indeed, we also observed the treatment of macrophages with Vpx + VLPs enhanced the ERT activity of HIV-1 BaL (Additional file 1: Figure S2). Collectively, the data presented in Fig. 3 suggests that the high ERT activity can be maintained in the viral particles produced from the macrophages with multiple exposures to Vpx that can maintain the high dNTP level during the long-term culture [21].

### Effect of dN treatment on HIV-1 ERT activity In macrophages

**Fig. 4:**
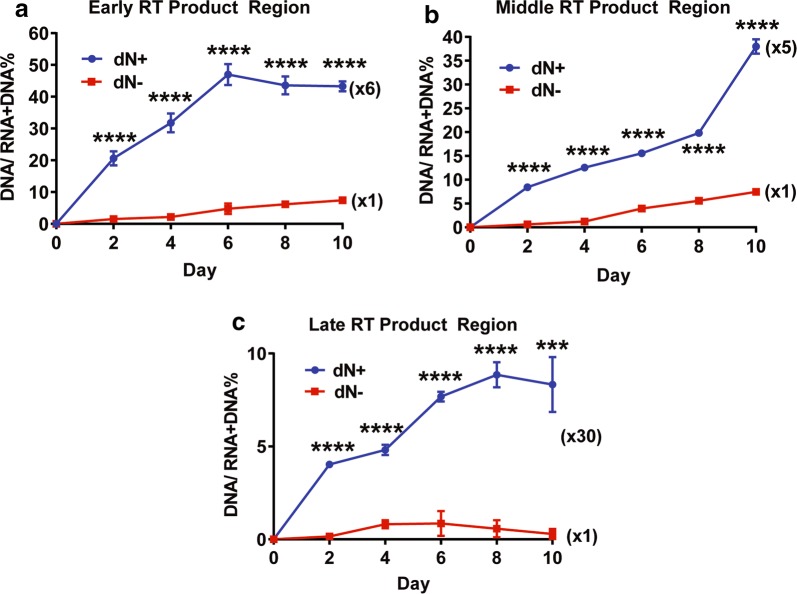
ERT activity comparison between HIV-1 89.6 viruses produced from primary macrophages with and without dN treatment. Primary macrophages were pre-treated with dNs (2.5 mM) for 3 h, and then infected the treated macrophages with an equal p24 level of dual tropic HIV-1 89.6 in triplicates. The remaining uninfected viruses were washed at 9 h post infection. The infected macrophages were cultured for 10 days post infection. The produced viruses were harvested at every 2 days, and total viral nucleic acids were extracted and used for measuring the ERT activity of the viral particles produced from dN (−) (red line) and dN (+) (blue line) treated macrophages for **a** early, **b** middle, and **c** late RT products. The data are the mean of three independent experiments with qPCR performed in duplicate, and error bars represent the standard error of the mean. ***P < 0.001; ****P < 0.0001

We previously reported that the treatment of nondividing myeloid cells with deoxynucleosides (dNs) elevates cellular dNTP concentration [22]. Next, we tested whether the dN treatment also enhances ERT activity of HIV-1 89.6. For this test, we pre-treated primary macrophages with 2.5 mM dNs, and we infect the macrophages with 89.6. The ERT activity of the produced viruses during 10-day culture were determined as described in Fig. 2. Indeed, as shown in Fig. 4, the dN treatment (dN +) enhanced the ERT activity of the produced HIV-1 89.6 in all three regions of the viral DNA by 5–30 folds, compared to the viruses produced from the dN untreated macrophages (dN-). This data demonstrates that, as observed with the Vpx treatment (Fig. 3), the dN treatment, which elevates cellular dNTP levels in macrophages, also promotes the ERT activity of HIV-1 in macrophages.

### Comparison of replication kinetics and Infectivity of HIV-1 89.6 produced from activated CD4^+^ T cells and macrophages with and without Vpx treatment

**Fig. 5:**
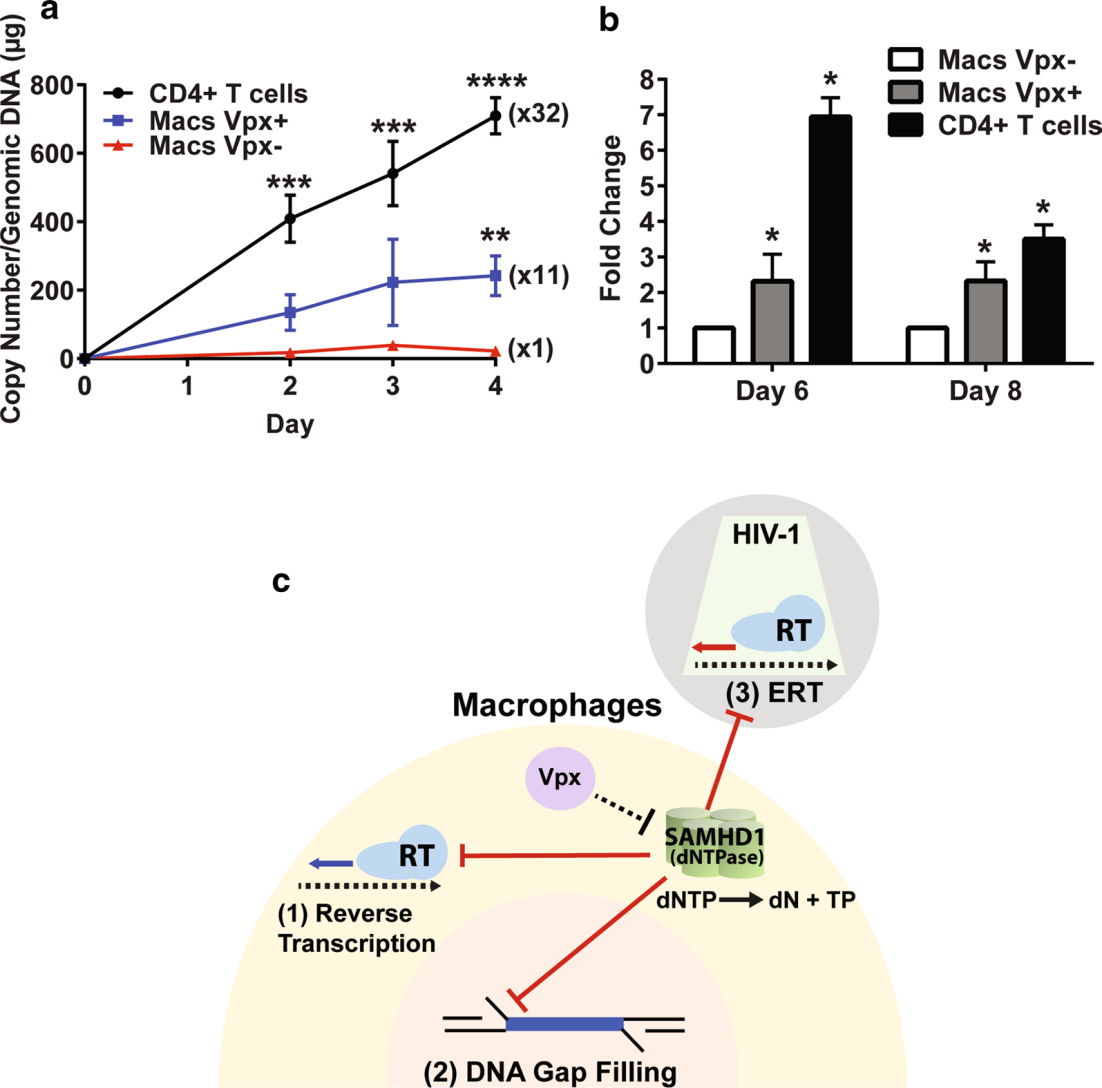
Reverse transcription kinetics and Infectivity comparisons of HIV-1 89.6 produced from macrophages treated with Vpx (−) VLPs and Vpx (+) VLPs, and activated CD4^+^ T cells, and three steps of HIV-1 replication cycle restricted by host SAMHD1 protein. Fresh macrophages from healthy donors were infected in triplicates with the HIV-1 89.6 viruses collected at day 8 from Vpx (−) VLP treated macrophages, Vpx (+) VLP treated macrophages (from Fig. 3) or collected at day 3 from the activated CD4^+^ T cells (see Fig. 2). **a** Reverse transcription kinetics of these three viruses were determined by measuring the copy numbers of 2LTR circle DNAs with the cellular genomic DNAs isolated from the infected cells. **b** Infectivity of the three different viruses was determined by measuring the viral RNA copy numbers (early RT products) at days 6 and 8 post infection. **c** Three steps of HIV-1 replication cycle restricted by host SAMHD1 in macrophages. SAMHD1 suppression against the three steps of HIV-1 cycle that consume dNTPs is marked as red lines. HIV-1 particles: grey, macrophage cytoplasm: yellow, and macrophages nucleus: pink. Reverse transcribed products by RT are marked as thick arrows. Red arrow: ERT product within the core (green) of produced viral particle, Blue arrow: reverse transcribed product in cytoplasm post infection. SAMHD1 tetramer depletes cellular dNTPs by hydrolyzing dNTP to dN and triphosphate (TP). Vpx (purple) counteracts SAMHD1. The data are the mean of three independent experiments with qPCR performed in duplicate, and error bars represent the standard error of the mean. *P < 0.05; **P < 0.01; ***P < 0.001; ****P < 0.0001

It was previously reported that the viruses with higher ERT activity have higher infectivity to non-dividing cells [20]. Next, we compared both reverse transcription rate and infectivity of the HIV-1 89.6 produced from macrophages treated with Vpx (−) and Vpx (+) VLPs as well as activated CD4^+^ T cells to fresh macrophages. For these comparisons, first, an equal p24 level of the viruses collected at day 8 from the VLP treated macrophages (Fig. 3) and also collected at days 3 from the activated CD4^+^ T cells (Fig. 2) were used to infect to fresh monocyte derived macrophages pooled from 5 new healthy donors, and we monitored the reverse transcription kinetics for 4 days post infection by measuring the copy number of 2LTR circle DNA, which is the completed reverse transcription product. As shown in Fig. 5a, HIV-1 89.6 produced from the Vpx (+) VLP treated macrophages displayed 11 times higher 2LTR circle DNA copy numbers, compared to the viruses from the Vpx (−) VLP treated macrophages. The viruses from the activated CD4^+^ T cells still showed faster reverse transcription kinetics, compared to the viruses from the Vpx (+) VLP treated macrophages. Next, we monitored viral production by determining viral RNA copy numbers of the produced viral particles in the collected media at days 6 and 8 post infection. As shown in Fig. 5b, the macrophages infected with the viruses from activated CD4^+^ T cells showed the highest viral production, and importantly, the viruses from the Vpx (+) VLP treated macrophages showed more viral production, compared to the viruses produced from the Vpx (−) VLP treated macrophages. Importantly, in these experiments, only a small volume of the harvested media, which contains the produced viruses, was used and diluted (1/80) for infecting the fresh macrophages. Therefore, it is unlikely that the observed high infectivity of the viruses produced from the Vpx (+) VLP treated macrophages is due to the carryover of the Vpx (+) VLPs contained in the harvested media. Indeed, we did not observe the SAMHD1 degradation in the fresh macrophages infected with the viruses harvested from the Vpx (+) treated macrophages (Additional file 1: Figure S3). Collectively, the data in Fig. 5a, b demonstrate that the Vpx, which enhances the ERT activity of the produced viruses, increases viral reverse transcription kinetics and infectivity in macrophages.

## Discussion

While HIV-1 replication is kinetically suppressed, HIV-2 and some SIV strains are able to rapidly replicate even in macrophages. Vpx of HIV-2 and some SIV strains (i.e. SIVsm) as well as Vpr of other SIV strains (i.e. SIVagm) [23] enables these lentiviruses to overcome the SAMHD1-mediated dNTP depletion in macrophages. So far, two major steps of lentivirus life cycle, reverse transcription and DNA gap filling during integration, were known to be affected by SAMHD1, and the Vpx-induced SAMHD1 degradation and subsequent dNTP level elevation can kinetically facilitate both reverse transcription and DNA gap filling specifically in macrophages. Now, the data presented in this study support that the higher ERT activity of HIV-2 may also contribute to the rapid replication kinetic of HIV-2 in macrophages.

The dNTP concentration found in macrophages is below the K_m_ values of HIV-1 RT, and therefore, the enzymatic proviral DNA synthesis rate of HIV-1 RT at the macrophage dNTP concentration is below the maximal rate of HIV-1 RT [6]. However, Vpx enhances the cellular dNTP concentration (Fig. 2a) above the K_m_ values of HIV-1 RT in macrophages, which can accelerate the reverse transcription kinetics in macrophages. Furthermore, our data the dNTP elevation by Vpx in macrophages enhances the ERT activity, which also elevate the infectivity of the viruses produced from the Vpx (+) VLP treated macrophages to fresh macrophages. Interestingly, the macrophages dNTP concentration enhanced by Vpx is still lower than the dNTP concentrations found in activated CD4^+^ T cells (3–5 μM, Fig. 2a) [6], and this high dNTP concentration found in activated CD4^+^ T cells contributed to the highest ERT activity and infectivity. It was previously reported that the early ERT product can occur independently of intracellular dNTP levels, instead by using exogenous dNTPs that could exist in the natural microenvironments [20]. However, our tissue culture experimental setting does not include the exogenous dNTPs. Overall, the data presented in this study support that the cellular dNTP concentrations in the virus-producing cells can contribute to the infectivity of the produced HIV-1 particles to macrophages by modulating the ERT activity of the produced viruses that consumes the dNTPs co-packaged during viral budding.

## Conclusions

Figure 5c illustrates three steps of HIV-1 replication cycle that are suppressed by the dNTPase activity of the host SAMHD1 protein because these three steps all consume dNTPs. First, the reverse transcription step was the first identified step of HIV-1 replication cycle in nondividing myeloid cells that host SAMHD1 restricts by reducing the cellular dNTP concentration [11, 12]. Second, since the ssDNA gap filling step of HIV-1 integration requires cellular dNTPs, the DNA gap filling step can be controlled by SAMHD1 [17, 18]. Finally, the data presented in this study identified the ERT step of HIV-1 as the third step of HIV-1 replication cycle that is restricted by host SAMHD1 in nondividing myeloid cells. Vpx, which counteracts SAMHD1, can derepress these three steps of HIV-1 replication cycle restricted by SAMHD1. In conclusion, this study suggests that SAMHD1 is a highly effective anti-HIV-1 restriction factor that can suppress three different steps of viral replication cycle by modulating cellular dNTP levels in nondividing myeloid cells.

## Methods

### Cells, viruses and virus like particles

Human primary macrophages were prepared by the GMCSF-mediated differentiation of monocytes isolated from buffy coats as previously described [24, 25]. Briefly, monocytes were isolated by positive selection (CD14 microbeads; Miltenyi Biotec) using an automated magnetic cell sorter (AutoMacs, Miltenyi Biotec) from human buffy coats of 5 healthy donors (LifeSouth Community Blood Center, Dunwoody, GA). Monocytes were pooled in an equal number per donor and then differentiated to macrophages with GMCSF (10 ng/ml, Miltenyl Biotec) for 7 days prior to use with all experiments. HIV-1 89.6 viruses were initially prepared from the transfection of HIV-1 89.6 molecular clone plasmid (kindly provided from NIH AIDS Reagent Program, Division of AIDS, NIAID, NIH, https://www.aidsreagent.org/reagentdetail.cfm?t=viruses&id=704) to 293 FT cells (Invitrogen). The produced viruses were propagated for 8 passages in CEMx174 cells until the beta-lactamase gene encoded in the plasmid was not detected in the PCR amplification in the viral nucleic acid extracted from the culture media; the absence of this gene demonstrated a plasmid-free culture. HIV-1 p24 ELISA (Advanced Bioscience Laboratories Inc.) was used for monitoring and quantitating the produced viruses. HIV-2 Rod was also prepared as described for HIV-1 89.6, and the viral production was monitored by p27 ELISA kit (Advanced Bioscience Laboratories Inc.). HIV-1 BaL was also obtained from NIH AIDS Reagent Program. Vpx (−) and Vpx (+) virus like particles (VLPs) were prepared as previously described [21]. Briefly, 293 FT cells were transfected with pSIV Vpx (−) or pSIV Vpx (+) and pVSV-G, and the media were collected in day 3. After removing the cells and other debris by low-speed centrifugation and filtration, the produced VLPs were collected by ultracentrifugation (28,000 rpm for 90 min). The pellets were re-suspended to serum free DMEM and aliquots were stored at − 80 °C.

### Assay for SAMHD1 degradation by Vpx

2 × 10^6^ macrophages were treated with Vpx (−) or Vpx (+) VLPs in 6 well plates, and the cell lysates were prepared with RIPA buffer at 24 h post treatment. Human SAMHD1 and GAPDH levels in the lysates were determined by western blots. Human anti-SAMHD1 (Abcam) and anti-GAPDH (Abcam) as well as secondary anti-Mouse and anti-Rabbit antibodies (GE Healthcare) were used for the visualization.

### Assay for cellular dNTP levels

dNTPs in 2 × 10^6^ macrophages treated with Vpx (−) or Vpx (+) VLPs for 24 h were extracted with 65% methanol, and the extracted dNTPs were determined by the primer extension based dNTP assay as previously described [6]. The amount of each of four dNTPs per 1 × 10^6^ cells as well as dNTP concentration was calculated for comparison.

### Assay for ERT activity of HIV-1

1 × 10^6^ macrophages pre-treated with Vpx (−) or Vpx (+) VLPs for 24 h and 2.5 mM dNs for 3 h and activated CD4^+^ T cells were infected in triplicates with an equal p24 level of HIV-1 89.6. After 9-h incubation, the remaining viruses were removed by washing the cells three times. The infected cells were cultured and the viruses in the media were collected at varying time points. The total viral nucleic acids were isolated by QIAGEN EZ1 virus mini kit v2.0. The regions of HIV-1 89.6 gene [26], early, mid and late regions, were chosen for the determination of viral RNA and DNA copy numbers in the isolated total viral nucleic acids. Primers for HIV-1 89.6 ERT assay: early region (559–643) primers: 5′-GTGCCCGTCTGTTGTGTGAC-3′ and 5′-GGGCGCCACTGCTAGAGATTT-3′, early region probe: 5′-CTAGAGATCCCTCAGACCATCC TAGTTAGTGTAG-3′. Middle region (8782–8928) primers: 5′-CTATAAGATGGGAGGCAAGTG G-3′ and 5′-CTTGTGATAGCTCCATGTCTCG-3′. Middle region probe: 5′-AAACGTAGGGCAG AGGGATGGC-3′. Late region (556–698) primers: 5′-TGTGTGCCCGTCTGTTGTGT-3′ and 5′-GAGTCCTGCGTCGAGAGATC-3′, late region probe: 5′-CAGTGGCGCCCGAACAGGGA-3′. Primers for HIV-2 Rod ERT assay: early region (225–309) primers: 5′-TCACCTGAGTAACAAGACCC-3′ and 5′-GGCGCCAACCTGCTAGG GATTT-3, early region probe: 5′-TTCTTGCTTTGG GAAACCGAGGC-3′. Middle region primers (8681–8780): 5′-TCCAAGAAGGATCAGACAGG-3′ and 5′-TCGCC CTCCTGTGAGGGACGG-3′, middle region probe: 5′-ATCGCCCTCCTGTGAG G-3′. Late region primers (241–352): 5′-ACCCTGGTCTGTTAGGACCC-3′ and 5′-GCCGTGTTC CAAGACTTCTCAG-3′, late region probe: 5′-TCCCTAGCATGGCGCCTG-3′. For both RNA and DNA copy determination, Q-RT PCR (LightCycler^®^ 480 RNA Master Hydrolysis Probes) was used, and, for DNA copy number, the same protocol and reagents were used except skipping the reverse transcription step of the PCR protocol with the inactivation of RT contained in the RT-PCR kits by preheating at 98 °C. p89.6 plasmid was used as a copy number control, and Q-RT PCR and Q-PCR were conducted in triplicates per viral nucleic acid sample. ERT activity was determined by ratio between DNA copy number and (RNA + DNA) copy number in a single viral nucleic acid sample extracted. The same primers and probes were used from the analysis of the early ERT activity of HIV-1 BaL. Middle region primers for BaL: 5′TGGGTTATGAACTCCATCCTGAT′3 and 5′TGTCATTGACAG TCCAGCGTCT′3, middle region probe 5′TTTCTGGC AGCACTATAGGCTGTACTGTCCATT.

### Quantitative 2LTR circle DNA PCR

Macrophages from 5 healthy donors were infected with an equal p24 amount of HIV-1 89.6 collected at day 4 from the Vpx (−) VLP or Vpx (+) VLP treated macrophages as well as at day 3 from the activated CD4^+^ T cells (Fig. 2) in triplicates. The infected cells (in triplicates) were collected and the total cellular genomic DNA was isolated from the cells (Wizard^®^ Genomic DNA Purification Kit), and used for quantitative HIV-1 2LTR circle DNA PCR. The protocol, primers and probe for the quantitative HIV-1 89.6 2LTR circle PCR were the same as the one previously used for NL4-3 [12].

### Viral production assay by Q-RT PCR

The viruses in the culture media of the infected cells (triplicates) were collected and the total nucleic acids were extracted from the viruses as described above. The collected viruses were quantitated by Q-RT PCR for the copy numbers of the early reverse transcription product in the extracted total viral nucleic acids as described above.

### Statistical analysis

Statistical analyses were performed with an unpaired t test (Figs. 1b–e, 3a–c, 4a–c) comparing each time point independently in each figure. The statistical analysis was also performed using an ordinary one-way analysis of variance (ANOVA) with multiple comparisons comparing each column to the Vpx- or NT control (Figs. 2a, c, 5a, b). Time point Days 3 and 4 were analyzed together in Fig. 2a. The statistical significance of differences between data is indicated as follows: *P < 0.05; **P < 0.01; ***P < 0.001; ****P < 0.0001.

## Additional file

**Additional file 1.** **Figure S1**: **(A)** Cellular concentrations of dGTP, dCTP and dTTP determined in the experiments described in Figure 2A. NT: No treatment control. See Figure 2 for statistic analysis. **(B)** SAMHD1 degradation in macrophages treated with Vpx (-) and Vpx (+) VLPs. NT: No treatment control. SAMHD1 was detected by anti-SAMHD1 antibody, and cellular GAPDH protein was used as a loading control. **Figure S2**: **Effect of Vpx on ERT activity of HIV-1 BaL**. The viral culture and ERT assay for HIV-1 BaL were conducted under the same condition described in Figure 3 for HIV-1 89.6. Early **(A)** and Middle **(B)** RT products were used for the ERT activity comparision. The data are the mean of three independent experiments with qPCR or dNTP assay performed in duplicate, and error bars represent the standard error of the mean. *,P<0.05; **,P<0.01; ***,P<0.001.** Figure S3**: **Stability of SAMHD1 post infection**. SAMHD1 levels were determined by western blot in fresh macrophages infected with the HIV-1 89.6 viruses collected at day 6 from Vpx (-) VLP treated macrophages and at day 8 from Vpx (+) VLP treated macrophages. GAPDH: Loading control. NT: fresh macrophage without treatment.

## Abbreviations

HIV-1: human immunodeficiency virus type 1
HIV-2: human immunodeficiency type 2
SIV: simian immunodeficiency virus
Vpx: viral protein X
SAMHD1: SAM domain and HD domain containing protein 1
RT: reverse transcriptase
ERT: endogenous reverse transcription
dNTP: deoxynucleoside triphosphate
VLP: virus like particles
