# Supplementary material for: Host SAMHD1 protein restricts endogenous reverse transcription of HIV-1 in nondividing macrophages
Source: Retrovirology. 2018 Oct 13;15:69. doi: 10.1186/s12977-018-0452-z (PMC6186296; doi:10.1186/s12977-018-0452-z)
Supplement: Supplementary file 1 — Additional file 1. Figure S1: (A) Cellular concentrations of dGTP, dCTP and dTTP determined in the experiments described in Figure 2A. NT: No treatment control. See Figure 2 for statistic analysis. (B) SAMHD1 degradation in macrophages treated with Vpx (-) and Vpx (+) VLPs. NT: No treatment control. SAMHD1 was detected by anti-SAMHD1 antibody, and cellular GAPDH protein was used as a loading control. Figure S2: Effect of Vpx on ERT activity of HIV-1 BaL. The viral culture and ERT assay for HIV-1 BaL were conducted under the same condition described in Figure 3 for HIV-1 89.6. Early (A) and Middle (B) RT products were used for the ERT activity comparision. The data are the mean of three independent experiments with qPCR or dNTP assay performed in duplicate, and error bars represent the standard error of the mean. *,P<0.05; **,P<0.01; ***,P<0.001. Figure S3: Stability of SAMHD1 post infection. SAMHD1 levels were determined by western blot in fresh macrophages infected with the HIV-1 89.6 viruses collected at day 6 from Vpx (-) VLP treated macrophages and at day 8 from Vpx (+) VLP treated macrophages. GAPDH: Loading control. NT: fresh macrophage without treatment. [file 12977_2018_452_MOESM1_ESM.pptx]

## Slide 1
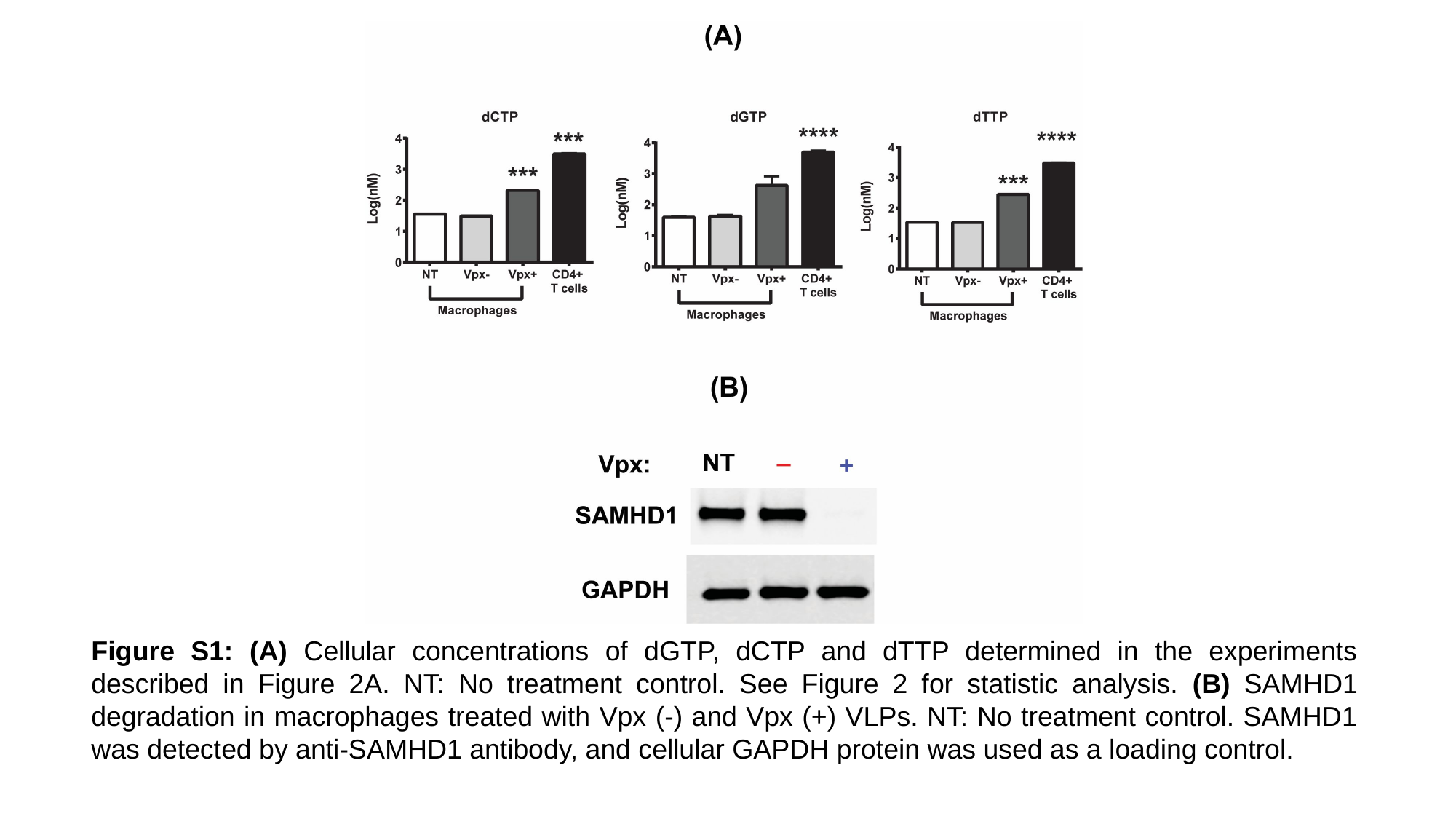

Figure S1: (A) Cellular concentrations of dGTP, dCTP and dTTP determined in the experiments described in Figure 2A. NT: No treatment control. See Figure 2 for statistic analysis. (B) SAMHD1 degradation in macrophages treated with Vpx (-) and Vpx (+) VLPs. NT: No treatment control. SAMHD1 was detected by anti-SAMHD1 antibody, and cellular GAPDH protein was used as a loading control.

## Slide 2
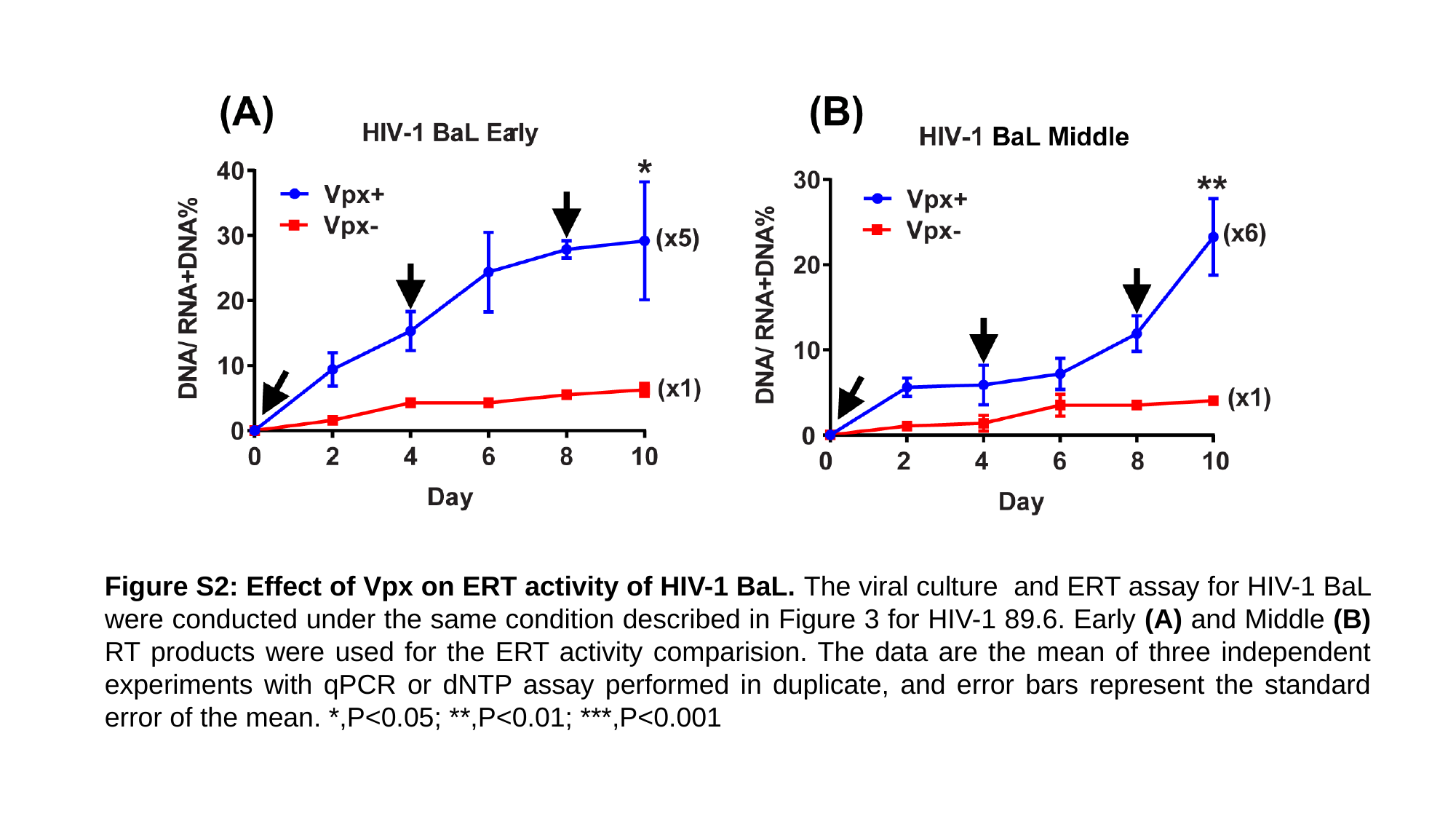

Figure S2: Effect of Vpx on ERT activity of HIV-1 BaL. The viral culture and ERT assay for HIV-1 BaL were conducted under the same condition described in Figure 3 for HIV-1 89.6. Early (A) and Middle (B) RT products were used for the ERT activity comparision. The data are the mean of three independent experiments with qPCR or dNTP assay performed in duplicate, and error bars represent the standard error of the mean. *,P<0.05; **,P<0.01; ***,P<0.001

## Slide 3
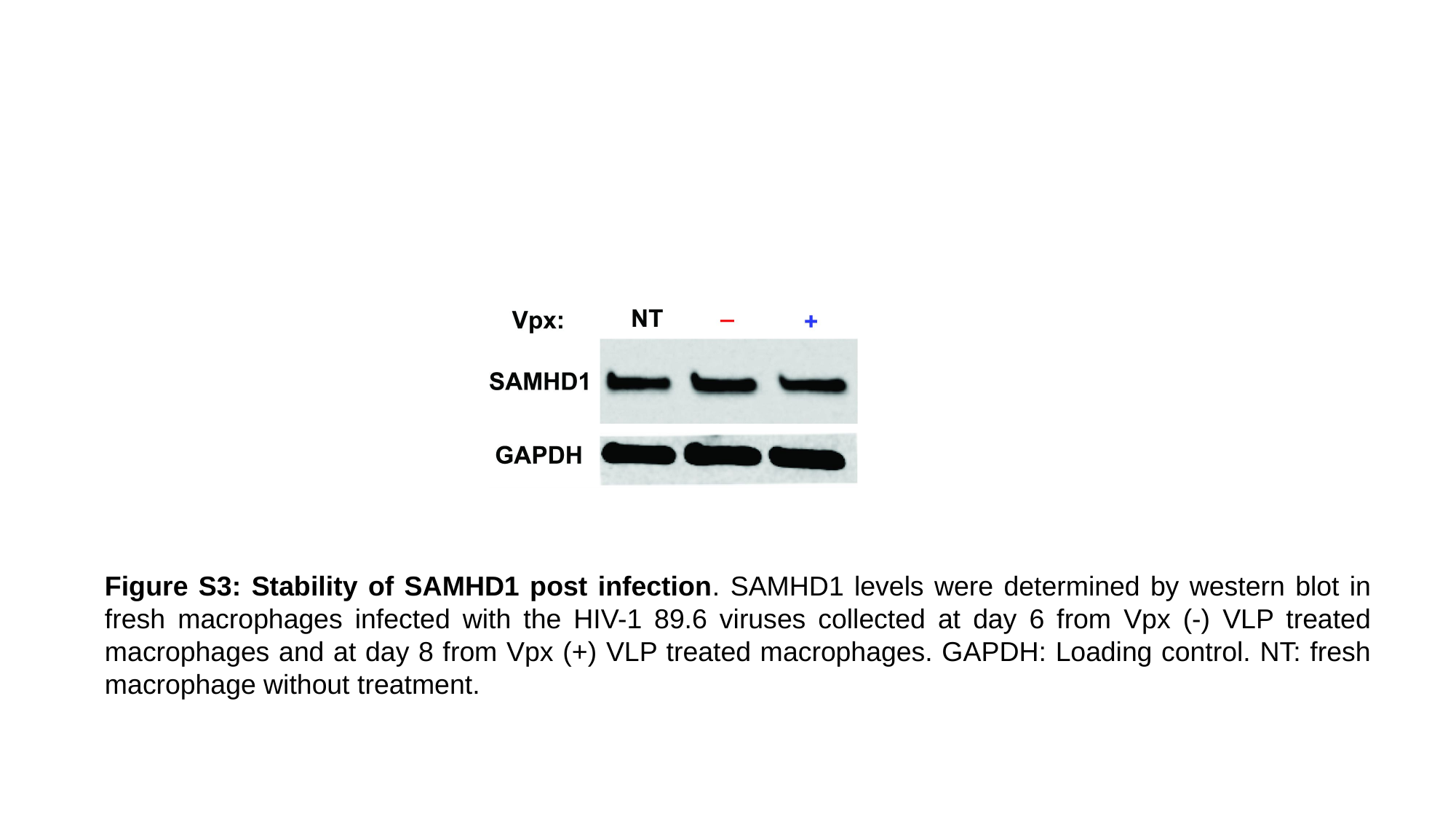

Figure S3: Stability of SAMHD1 post infection. SAMHD1 levels were determined by western blot in fresh macrophages infected with the HIV-1 89.6 viruses collected at day 6 from Vpx (-) VLP treated macrophages and at day 8 from Vpx (+) VLP treated macrophages. GAPDH: Loading control. NT: fresh macrophage without treatment.
